# The complete chloroplast genome sequence of *Echinocodon Lobophyllus* (Campanulaceae) revealed by next-generation sequencing and phylogenetic implication

**DOI:** 10.1080/23802359.2018.1462117

**Published:** 2018-04-10

**Authors:** Yu Chao Wang, Ya Fu Zhou, Chen Chen, Shao Li Mao

**Affiliations:** Xi’an Botanical Garden of Shaanxi Province/Institute of Botany of Shaanxi Province, Xi’an, China

**Keywords:** *Echinocodon lobophyllus*, chloroplast genome, Endangered species, phylogenetic relationship

## Abstract

The complete chloroplast genome sequence of *Echinocodon lobophyllus* was determined by Illumina pair-end sequencing. The results showed that the complete plastid genome was 169,419 bp in length, containing a large single copy (LSC) of 85,599 bp and a small single copy (SSC) of 8054 bp, which were separated by a pair of 37,883 bp inverted repeats (IRs). A total of 109 unique genes were annotated, including 75 protein coding genes, 30 tRNA genes, and 4 rRNA genes. Among these genes, 16 genes contained one or two introns. The overall GC contents of the plastid genome were 38.4%. A maximum likelihood phylogenetic analysis showed that *E. lobophyllus* and *Codonopsis minima* formed one clade at the base of the phylogenetic tree of Campanulaceae with a high support value.

*Echinocodon lobophyllus* Hong was published as a new species and genus by Hong Deyuan (Hong 1984). It was found only in a small ecological zone of NW Hubei and SE Shaanxi, China, meanwhile, it is also a rare and Endangered species in higher plant (Hong 2014). In this paper, the complete chloroplast genome sequence of *E*. *lobophyllus* was detected by Illumina pair-end sequencing, with the aim to provide new data for study and conservation of the only species in *Echinocodon*. The annotated chloroplast genome sequence of this species has been submitted to GenBank with the accession number MH029157.

The plant material was sampled from Mt. Qinling, Shaanxi, China (32°53′54″N, 109°52′44″E). The voucher specimen was deposited in Institute of Botany of Shaanxi Province. The complete chloroplast genome of *E*. *lobophyllus* was sequenced on the Illumina Hiseq 2500 sequencing platform (Illumina, CA, USA) and produced 28,824,522 raw reads. The raw paired-end reads were filtered by using CLC Genomics Workbench 8 (CLC Bio, Aarhus, Denmark), and were then aligned to the *Codonopsis minima* chloroplast genome sequence (KY587457) as a reference using MITObim v1.8 (GitHub, San Francisco, USA) (Hahn et al. 2013) and Mira 4.0.2 (Chevreux et al. 2004) to assembly. The whole chloroplast genome sequence was annotated using the software Geneious v 10.1.2 (Biomatters Ltd., Auckland, New Zealand).

The results showed the complete chloroplast genome of *E. lobophyllus* was 169,419 bp in length, which contained a large single copy (LSC) region of 85,599 bp, a small single copy (SSC) region of 8054 bp, and a pair of inverted repeat (IR) regions of 37,883 bp. The plastid genome contained a total of 109 unique genes constituting 75 protein coding genes, 30 transfer RNA (tRNA) genes, and 4 ribosomal RNA (rRNA) genes. In addition, there were 22 genes (11 protein coding genes, 7 tRNA genes, and 4 rRNA genes) duplicated in the IR region. Among the 109 unique genes, 10 protein coding genes (*atpF*, *clpP*, *ndhA*, *ndhB*, *petB*, *petD*, *rpl2*, *rpl16*, *rps12*, *ycf3*) and six transfer RNA (*trnG-UCC*, *trnK-UUU*, *trnV-UAC*, *trnL-UAA*, *trnI-GAU*, *trnA-UGC*) contained one or two introns. The *rps12* gene was a trans-spliced gene with a 5′ end exon in the LSC region and two duplicated 3′ end exons in the IR regions. The overall GC content was 38.4%, and in the regions of IR, LSC and SSC were 40.5, 33.1, and 31.7%, respectively.

Phylogenetic analysis was performed using RAxML (Stamatakis 2014) based on 42 protein coding genes (26,901 bp) of eight species in Campanulaceae and two outgroups (*Helwingia himalaica* and *Ilex pubescens*). The result revealed that *E. lobophyllus* and *C. minima* formed one clade with a high bootstrap value at the base of the phylogenetic tree (Figure 1).

**Figure 1.**
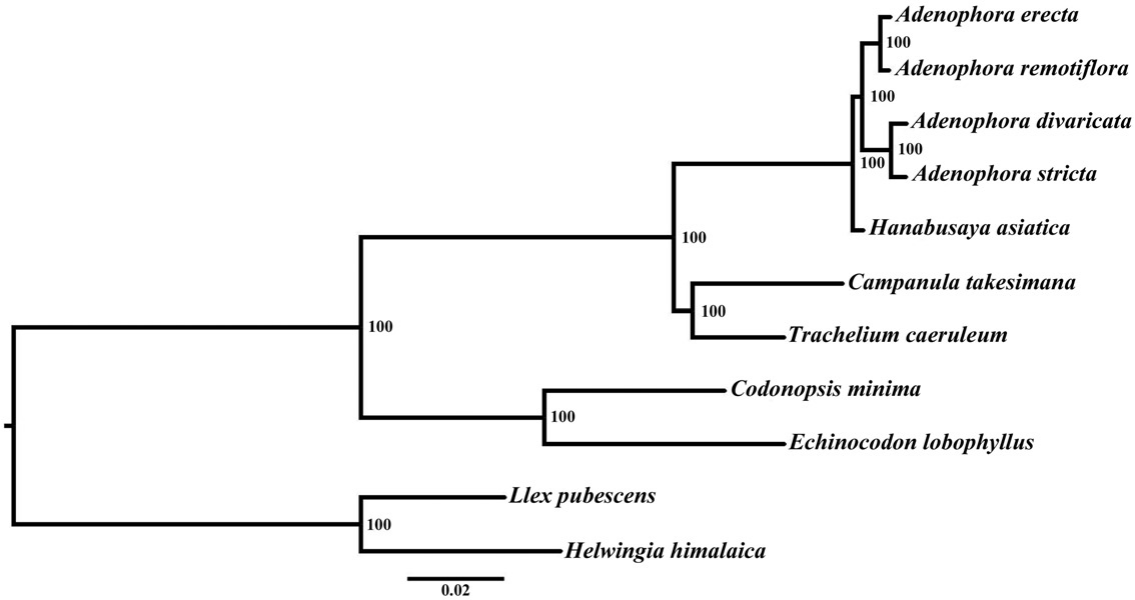
Phylogenetic relationships of Campanulaceae using 42 PCGs concatenated dataset.
